# Patient Characteristics Associated with Tuberculosis Treatment Default: A Cohort Study in a High-Incidence Area of Lima, Peru

**DOI:** 10.1371/journal.pone.0128541

**Published:** 2015-06-05

**Authors:** Brian Lackey, Carlos Seas, Patrick Van der Stuyft, Larissa Otero

**Affiliations:** 1 University of Texas School of Public Health Austin Regional Campus, Austin, Texas, United States of America; 2 Instituto de Medicina Tropical Alexander von Humboldt, Universidad Peruana Cayetano Heredia, Lima, Peru; 3 Facultad de Medicina Alberto Hurtado, Universidad Peruana Cayetano Heredia, Lima, Peru; 4 General Epidemiology and Disease Control Unit, Department of Public Health, Institute of Tropical Medicine, Antwerp, Belgium; 5 Department of Public Health, Faculty of Medicine, Ghent University, Ghent, Belgium; National HIV and Retrovirology Laboratories, CANADA

## Abstract

**Background:**

Although tuberculosis (TB) is usually curable with antibiotics, poor adherence to medication can lead to increased transmission, drug resistance, and death. Prior research has shown several factors to be associated with poor adherence, but this problem remains a substantial barrier to global TB control. We studied patients in a high-incidence district of Lima, Peru to identify factors associated with premature termination of treatment (treatment default).

**Methods:**

We conducted a prospective cohort study of adult smear-positive TB patients enrolled between January 2010 and December 2011 with no history of TB disease. Descriptive statistics and multivariable logistic regression analyses were performed to determine risk factors associated with treatment default.

**Results:**

Of the 1233 patients studied, 127 (10%) defaulted from treatment. Patients who defaulted were more likely to have used illegal drugs (OR = 4.78, 95% CI: 3.05-7.49), have multidrug-resistant TB (OR = 3.04, 95% CI: 1.58-5.85), not have been tested for HIV (OR = 2.30, 95% CI: 1.50-3.54), drink alcohol at least weekly (OR = 2.22, 95% CI: 1.40-3.52), be underweight (OR = 2.08, 95% CI: 1.21-3.56), or not have completed secondary education (OR = 1.55, 95% CI: 1.03-2.33).

**Conclusions:**

Our study identified several factors associated with defaulting from treatment, suggesting a complex set of causes that might lead to default. Addressing these factors individually would be difficult, but they might help to identify certain high-risk patients for supplemental intervention prior to treatment interruption. Treatment adherence remains a barrier to successful TB care and reducing the frequency of default is important for both the patients’ health and the health of the community.

## Introduction

In a review of medical treatment interventions, Haynes et al. posited that improving treatment adherence “may have a far greater impact on the health of the population than any improvement in specific medical treatments” [1]. Most research on medication adherence pertains to chronic diseases, since medication is taken for an extended period of time. However, some infectious diseases such as tuberculosis (TB) also require long medication regimens [2]. Treatment for drug-susceptible TB consists of a two-month intensive phase of daily medication followed by a four-month continuation phase of varying frequency, while treatment for multidrug-resistant TB (MDR-TB) can take substantially longer [3]. Low adherence to anti-TB medication is common and can lead to death, drug resistance, continued transmission in the community, and increased health system costs [4–6].

To ensure long-term treatment adherence, patients historically were isolated in TB sanatoria where they were provided treatment and kept separate from non-diseased individuals. However, studies performed in Madras, India in the late 1950s showed that this held no advantage over treatment at home for either the patient or family and sanatoria were largely eliminated as a TB control strategy [7–10]. Unfortunately, researchers began to find that patients did not always take medication as prescribed and direct observation of treatment began to be considered as a strategy for enforcing medication adherence for the duration of a treatment regimen [11–13]. Direct observation of each dose of medication has been the international standard since the World Health Organization (WHO) adopted it as part of the Directly Observed Therapy Short-course (DOTS) strategy in the early 1990s [14]. However, a systematic review recently found no empirical evidence of improvement over self administration of therapy [15]. Regardless, TB treatment adherence remains an issue even under directly observed therapy, with cure rates falling well below the global target of 90% [16].

Numerous papers have explored potential causes of TB treatment default (premature termination of treatment) in settings with directly observed therapy. Previous quantitative studies in other high incidence urban settings have found alcohol abuse, recreational drug use, male gender, previous treatment default, and unemployment to be associated with default from treatment [17,18]. A systematic review of qualitative research found that reasons for default generally fit into 8 specific categories: “organisation of treatment and care; interpretations of illness and wellness; the financial burden of treatment; knowledge, attitudes, and beliefs about treatment; law and immigration; personal characteristics and adherence behaviour; side effects; and family, community, and household support” [19].

Although it was an early adopter of DOTS as a TB control strategy, Peru continues to face a high burden of TB, with an incidence of 101 cases per 100,000 people as of 2011 [20]. While 5.3% of new TB cases are MDR, fully 24% of re-treated cases are classified as such [20], demonstrating the importance of successful treatment completion. Our primary objectives were to describe the frequency of treatment default in a high-incidence district of Northeast Lima and to identify factors associated with default in order to provide insight for policy makers and clinicians in improving care. We conducted a cohort study of new adult smear-positive cases of pulmonary TB in the district, looking for factors associated with treatment default.

## Methods

### Setting

The study was carried out in a semi-urban district of Northeastern Lima, Peru with a population of approximately one million people. The district had an annual TB incidence of 213 per 100,000 residents, with seven percent of new cases classified as MDR-TB [21]. The majority of TB cases in the district are managed at the 34 public health facilities, composed of 33 community health clinics and one hospital. The National Tuberculosis Program has a designated office in each of these facilities, where a nurse, a nurse aid, and supervising physician are responsible for the detection, diagnosis, and follow up of TB suspects. Cases of TB are treated through health center-based DOTS, with treatment provided free of cost to the patient.

### Study Design

We performed a prospective cohort study among adults with a first episode of smear-positive, pulmonary TB. Patients were enrolled between January 2010 and December 2011 and followed up until treatment completion, death, or transfer to another jurisdiction. Trained field workers visited the health centers daily and invited patients to participate in the study upon diagnosis of smear-positive, pulmonary TB. Upon enrollment, field workers interviewed each patient to collect demographic, social, and medical information with a structured questionnaire. They did not interfere with routine patient management and treatment was carried out according to standard Ministry of Health protocols. We used a locally developed and validated scale to determine socioeconomic status [22]. HIV screening is an opt-out component of the national TB program and results were abstracted from patients’ files if available. Treatment outcome was obtained from patients’ charts.

### Data Management and Analysis

Data were entered by single entry into a Microsoft Access database (Microsoft Corporation, Redmond, WA, US). We checked the accuracy of variables for 10% of study subjects, reviewing 100% for a variable if mistakes were found in more than 10% of reviewed files. Where categorization was not implicit, potential predictor variables were classified as follows. Alcohol use was collected in 7 categories of increasing frequency, but dichotomized for analysis as at least weekly or not, based on a strong natural break in patient data. HIV status was classified as positive, negative, or “test was not done.” Treatment outcomes were defined according to standard WHO definitions, with treatment default defined as two months or more of continuously missed doses [3]. We compared patients who defaulted from treatment with those of all other outcomes. However, if any variables were associated with both treatment default and death or another negative outcome, this dichotomization could bias resulting odds ratios (OR) toward the null. Therefore additional analyses were conducted comparing those defaulting from treatment with only those who completed treatment, as done by Franke et al. in a similar situation previously [4]. Statistical analysis was performed using Stata 12.1 for Mac (Stata Corp., College Station, TX, USA). Potential predictor variables were analyzed in bivariate with Chi-square tests and OR with 95% confidence intervals (CI). The independent effect of each potential predictor variable was determined using a multivariable logistic regression model with a backward fitting variable selection algorithm. All predictor variables associated with the outcome at a p value of <0.2 were included in the original model.

### Ethical Considerations

The study protocol was approved by the Institutional Review Board of Universidad Peruana Cayetano Heredia and the East Lima DISA (regional health authority). All patients provided written informed consent and received a copy of the form. Data were managed securely and anonymously. Patients were treated at facilities of the Ministry of Health, which covered the cost of TB diagnosis and treatment.

## Results

### Subject Inclusion

**Fig 1:**
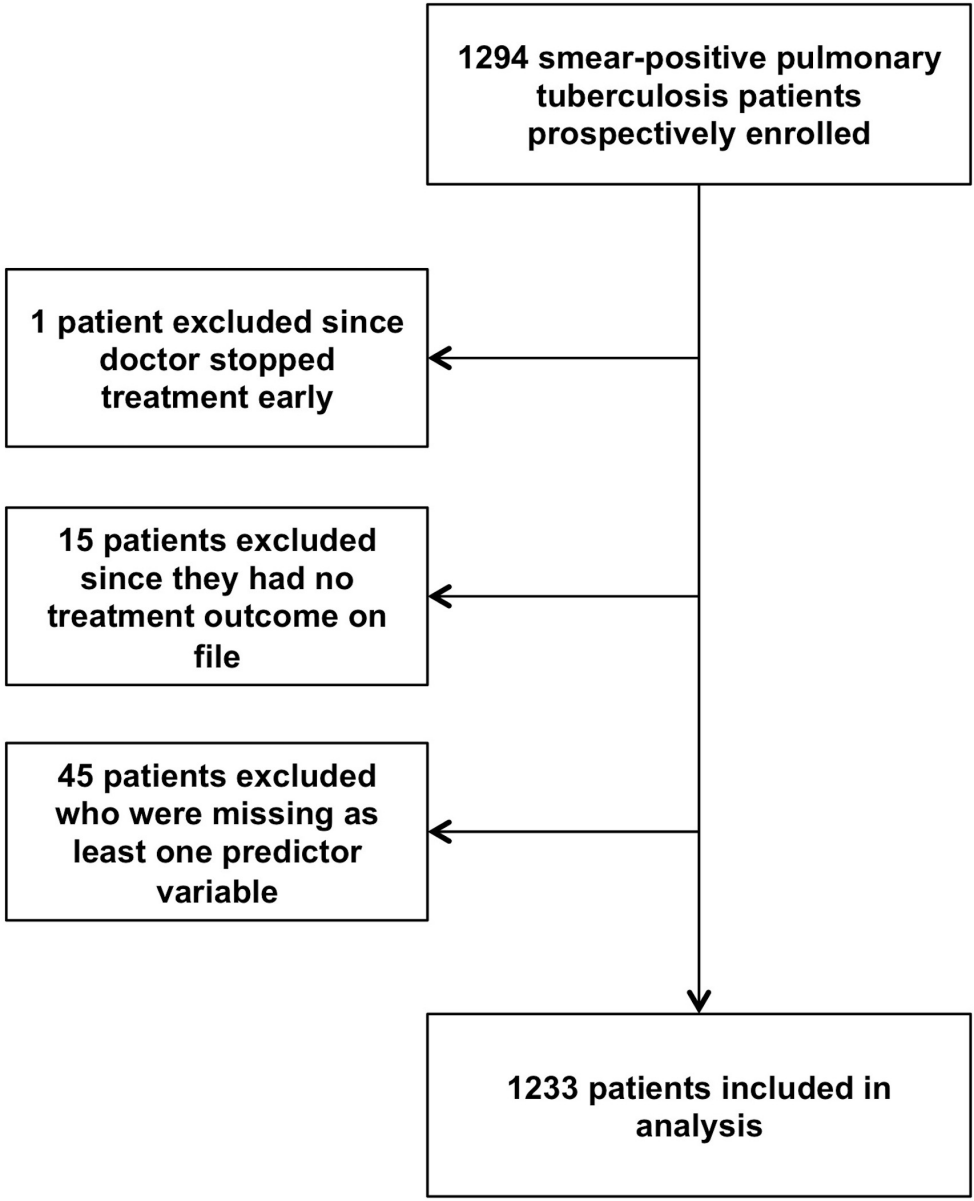
Flow of cohort study participants. We initially enrolled 1294 pulmonary, smear-positive tuberculosis patients into the study. One patient was excluded since the doctor stopped treatment early. Fifteen patients were excluded since there was no record of their treatment outcome on file. We excluded 45 patients from the analysis who were missing at least one predictor variable. Of the initial 1294 patients enrolled, 1233 remained for analysis.

Fig 1 shows the flow of participants in the study. During 2010 and 2011, a total of 1294 patients were enrolled. One patient’s treatment was stopped by direction of medical staff, while 15 (1%) had no record of outcome and were excluded from the analysis. An additional 45 (3%) were missing values for independent variables and were excluded, leaving 1233 subjects in the final analyses.

### Missing data

Data were missing most frequently for socioeconomic status (2%, n = 23), with less than 2% missing data for all other independent variables. Of the 1278 subjects with a treatment outcome on record, 1233 (96%) had full data for the variables included in the original multivariable model. As such, missing data were dealt with in analyses by complete case analysis.

### Characteristics of the study population

Baseline socio-demographic and medical characteristics of the 1233 remaining study subjects can be found in Table 1.

**Table 1:** Characteristics of 1,233 smear-positive pulmonary tuberculosis patients.

Variables	Patients (%)
**Age**	
Median years, IQR[Interquartile range]	26, IQR[21–37]
**Sex**	
Male	743 (60.3%)
Female	490 (39.7%)
**Marital Status**	
Married/cohabitating	469 (38.0%)
Divorced/separated	91 (7.4%)
Single	637 (51.7%)
Widowed	36 (2.9%)
**Socioeconomic Status**	
Not in poverty	1024 (83.0%)
Poverty or extreme poverty	209 (17.0%)
**Prison History**	
No	1178 (95.5%)
Yes	55 (4.5%)
**Completed Secondary School**	
No	514 (41.7%)
Yes	719 (58.3%)
**Smoking**	
Never smoked	742 (60.2%)
Currently smokes	51 (4.1%)
Used to smoke	440 (35.7%)
**Alcohol Use at Least Once Per Week**	
No	1000 (81.1%)
Yes	233 (18.9%)
**Drug Use**	
No	1043 (84.6%)
Yes	190 (15.4%)
**Rehab History**	
No	1156 (93.8%)
Yes	77 (6.2%)
**MDR Treatment Status**	
Standard Treatment	1152 (93.4%)
MDR Treatment	81 (6.6%)
**Body Mass Index (BMI)**	
Normal (18.5 to 24.9)	916 (74.3%)
Underweight (<18.5)	151 (12.2%)
Overweight/Obese (25 or greater)	166 (13.5%)
**Chronic Disease**	
No	1198 (97.2%)
Yes	35 (2.8%)
**HIV Status**	
Negative	916 (74.3%)
Positive	22 (1.8%)
Test not done	295 (23.9%)
**Diabetes**	
No	1179 (95.6%)
Yes	54 (4.4%)

### Treatment outcome

At time of analysis, 20 of the 1233 people (2%) were still on treatment, 1016 (82%) patients had completed treatment, and 40 (3%) had transferred out of the district. Thirty patients (2%) died during treatment. A total of 127 (10%) patients defaulted from treatment during the study period. Of these, 119 had a record of date of default, as noted by the Ministry of Health clinic. Among these, 19 (16%) defaulted during the first 60 days after treatment initiation, whereas 100 (84%) defaulted after this first stage of treatment.

### Risk factors for treatment default

Baseline alcohol use of at least once per week, low BMI at enrollment, lifetime history of illicit drug use, less than complete secondary education, treatment for MDR-TB, history of incarceration, history of residence in a rehabilitation center, poverty or extreme poverty, male sex, current or past smoking, and not having an HIV screening test done were all associated with defaulting from treatment in bivariate analysis. Furthermore, being in the oldest age quartile and having diabetes were both associated with a decreased risk of default in bivariate (see Table 2). After multivariable logistic regression analysis, only drug use, a BMI of less than 18.5, MDR treatment, alcohol use of at least once per week, not having been tested for HIV, and not completing secondary education could predict a higher rate of default (see Table 2). Since none of the diabetics defaulted from treatment, diabetes as a predictor variable was excluded from the regression analyses. When we excluded patients who had died, had transferred out, or who were still on treatment, and compared those who defaulted only to those who completed treatment, the multivariable output consisted of the same predictor variables. Only two significant variables had odds ratios that differed by more than 10%. The OR for low BMI (OR = 2.35, 95% CI: 1.35–4.07) increased by 13% and the odds ratio for MDR (OR = 4.49, 95% CI: 2.22–9.09) increased by 48%.

**Table 2:** Risk factors for default from anti-tuberculosis (TB) treatment regimens compared to all other outcomes, n = 1,233.

Variable		Patients	N Default (%)	Bivariate Analysis		Multivariable Analysis	
				Odds Ratio (95% CI)	P	Odds Ratio (95% CI)	P
**Age**					**.006**		
	21 and younger	321	37 (11.5%)	1.00			
	22 to 26	326	36 (11.0%)	0.95 (0.59–1.55)			
	27 to 37	291	39 (13.4%)	1.19 (0.73–1.92)			
	38 and older	295	15 (5.1%)	**0.41 (0.22–0.77)**			
**Sex**					**< .001**		
	Female	490	29 (5.9%)	1.00			
	Male	743	98 (13.2%)	**2.42 (1.57–3.72)**			
**Marital Status**					.681		
	Married/cohabitating	469	45 (9.6%)	1.00			
	Divorced/separated	91	10 (11.0%)	1.16 (0.56–2.40)			
	Single	637	70 (11.0%)	1.16 (0.78–1.73)			
	Widowed	36	2 (5.6%)	0.55 (0.13–2.38)			
**Socioeconomic Status**					**.034**		
	Not in poverty	1024	97 (9.5%)	1.00			
	Poverty/extreme poverty	209	30 (14.4%)	**1.60 (1.03–2.49)**			
**Prison History**					**<. 001**		
	No	1178	109 (9.3%)	1.00			
	Yes	55	18 (32.7%)	**4.77 (2.63–8.67)**			
**Completed secondary education**					**<. 001**		**.035**
	Yes	719	52 (7.2%)	1.00		1.00	
	No	514	75 (14.6%)	**2.19 (1.51–3.18)**		**1.55 (1.03–2.33)**	
**Smoking**					**<. 001**		
	Never smoked	742	50 (6.7%)	1.00			
	Currently smokes	51	8 (15.7%)	**2.57 (1.15–5.77)**			
	Used to smoke	440	69 (15.7%)	**2.57 (1.75–3.78)**			
**At Least Weekly Alcohol Use**					**<. 001**		**<. 001**
	No	1000	72 (7.2%)	1.00		1.00	
	Yes	233	55 (23.6%)	**3.98 (2.71–5.86)**		**2.22 (1.40–3.52)**	
**Drug Use**					**<. 001**		**<. 001**
	No	1043	64 (6.1%)	1.00		1.00	
	Yes	190	63 (33.2%)	**7.59 (5.12–11.25)**		**4.78 (3.05–7.49)**	
**Rehab History**					**<. 001**		
	No	1156	102 (8.8%)	1.00			
	Yes	77	25 (32.5%)	**4.97 (2.96–8.34)**			
**MDR Treatment**					**.004**		**<. 001**
	Standard Treatment	1152	111 (9.6%)	1.00		1.00	
	MDR Treatment	81	16 (19.8%)	**2.31 (1.29–4.13)**		**3.04 (1.58–5.85)**	
**Body Mass Index (BMI)**					**.011**		**.020**
	Normal (18.5 to 24.9)	916	91 (9.9%)	1.00		1.00	
	Underweight (<18.5)	151	25 (16.6%)	**1.80 (1.11–2.91)**		**2.08 (1.21–3.56)**	
	Overweight/Obese (> = 25)	166	11 (6.6%)	0.64 (0.34–1.23)		0.88 (0.44–1.73)	
**Other Chronic Disease**					.365		
	No	1198	125 (10.4%)	1.00			
	Yes	35	2 (5.7%)	0.52 (0.12–2.19)			
**HIV Status**					**<. 001**		**<. 001**
	Negative	916	72 (7.9%)	1.00		1.00	
	Positive	22	4 (18.2%)	2.60 (0.86–7.90)		1.39 (0.42–4.65)	
	Test not done	295	51 (17.3%)	**2.45 (1.67–3.60)**		**2.30 (1.50–3.54)**	
**Diabetes**					**.011**		
	No	1179	127 (10.8%)	1.00			
	Yes	54	0 (0.0%)	**.10 (0.00–0.56)** ^**a**^			

^a^ Odds ratio for diabetes variable was estimated using an exact logistic regression. Due to the perfect prediction for default, it was left out of the multivariable model variable selection process.

## Discussion

This study was carried out to quantify and explain the levels of treatment default in a high-incidence neighborhood of Lima, Peru. We found that about ten percent of patients prematurely defaulted from treatment, a figure lower than in some low-income settings, but still higher than ideal [6]. The large majority of these patients defaulted after completing the intensive phase of treatment, a pattern found in other studies as well [23]. We found that several factors were independently associated with default among patients, including substance use and treatment for MDR-TB. However, the number of significant factors identified is indicative of a complex combination of independent causes that might affect treatment adherence.

In our study, the strongest determinant of treatment default was illicit drug use. Similarly, frequent alcohol use was found to be a strong risk factor. These associations have been found in many other studies of TB treatment default [4,18,24,25]. While male sex and prison or rehab history were found to be associated with default in bivariate analysis, they are accounted for by drug and alcohol use and other variables in the multivariable model. It is possible that substance abuse could directly cause a patient to default from treatment, but we believe it is more likely that underlying behavioral differences or health system barriers unique to this population—and probably a combination of both—led to this high frequency of poor treatment adherence. Eliminating, or even reducing, rates of substance abuse in a population in this case might not lead to any change in default rates, even if such a task were feasible, but future research should look into the specific barriers to treatment success that this population faces so that interventions could be designed to address these concerns.

Whereas drug and alcohol use are frequently found in the literature to be associated with a higher risk of default, we did not find any other studies that linked a low BMI with a higher risk for treatment default. A review of treatment outcomes for MDR-TB patients in several countries showed a BMI under 18.5 to be associated with poor treatment outcomes including death, but was not significantly associated with defaulting from treatment [26]. Perhaps a patient with a low BMI would be more likely to have a severe comorbidity or be malnourished. Our study attempts to control for the major chronic comorbidities, though BMI could account for residual effects not found in the self-reported responses.

The fact that having no HIV test done was associated with defaulting from treatment is probably not generalizable beyond Lima. If not having been tested was randomly distributed across patients, then this group should be no more likely to default. This finding is indicative that tests are not being done for a systematic reason. We suggest that the local health authorities look into this discrepancy and work to test a higher proportion of TB patients for HIV co-infection.

Additionally, we found that patients who underwent treatment for MDR-TB were about three times more likely to default from treatment than those on the standard treatment regimen. Second-line medications are considerably more toxic than the standard medications, so it is possible that more severe adverse effects lead to this higher rate of default [27]. However, those on treatment for MDR-TB are also treated for a longer duration and are therefore given a longer timeframe in which to default. Either of these explanations, but likely a combination of the two, could explain the increased risk of default among patients being treated for MDR-TB.

Previous studies have shown mixed results regarding education and TB treatment adherence [18,28,29]. We found that failing to complete secondary education was associated with defaulting from the treatment regimen, though only moderately. This could indicate that our study had more power to detect a true association or that the relationship is modified by certain systemic or population characteristics.

Although diabetes was not able to be included in the multivariable model, those with diabetes appear to be less likely to default. It is possible that those patients on treatment for diabetes are already in the habit of taking daily medication or simply more comfortable with the healthcare system [30–32]. To learn more about this relationship and any potential explanations, future studies could look at preexisting medication routines and their effects on adherence to TB treatment.

Our study was limited by the self-reporting of several variables, which could reduce its accuracy. However, the local TB program will rely on self-reported measures in their work, so our results would be relevant for programmatic use. In addition, the study only included smear-positive TB patients, so these findings might not be representative of all cases of TB in the area. Furthermore, 15 of the patients had no record of treatment at the Ministry of Health facilities upon follow up, though we enrolled each of them after treatment initiation at the health facility. These missing outcomes are not likely to change the outcomes of the study, but it is nevertheless something to explore and prevent in the future. Moreover, we faced the issue of working within a relatively homogeneous system of clinics and were unable to explore any potential systemic factors that might predict default such as clinic-based therapy or clinic hours.

The results of this study are telling of a complex underlying set of determinants leading to treatment default. Additionally, even though characteristics like drug use were highly associated with default, many patients with several risk factors were able to complete treatment successfully. Future research should look into other elements that could explain these differences among patients with similar risk factors, particularly regarding patient beliefs and health system-related factors such as location of directly observed therapy, ease of travel to healthcare facilities, and clinic hours and wait times. Many of the factors we found will not lead directly to simple interventions, since modifying patient characteristics like education levels and substance use is likely outside the scope of TB control programs. However, our findings have the potential to help clinics identify patients at high risk for treatment default at time of diagnosis and design interventions to reduce barriers to successful treatment for these specific populations.
